# DRL-Driven Intelligent SFC Deployment in MEC Workload for Dynamic IoT Networks

**DOI:** 10.3390/s25144257

**Published:** 2025-07-08

**Authors:** Seyha Ros, Intae Ryoo, Seokhoon Kim

**Affiliations:** 1 Department of Software Convergence, Soonchunhyang University, Asan 31538, Republic of Korea; rosseyha003@gmail.com; 2 Department of Computer Engineering, Kyung Hee University, Yongin-si 17104, Republic of Korea; 3 Department of Computer Software Engineering, Soonchunhyang University, Asan 31538, Republic of Korea

**Keywords:** deep reinforcement learning, multi-access edge computing, network functions virtualization, service function chaining, internet of things

## Abstract

The rapid increase in the deployment of Internet of Things (IoT) sensor networks has led to an exponential growth in data generation and an unprecedented demand for efficient resource management infrastructure. Ensuring end-to-end communication across multiple heterogeneous network domains is crucial to maintaining Quality of Service (QoS) requirements, such as low latency and high computational capacity, for IoT applications. However, limited computing resources at multi-access edge computing (MEC), coupled with increasing IoT network requests during task offloading, often lead to network congestion, service latency, and inefficient resource utilization, degrading overall system performance. This paper proposes an intelligent task offloading and resource orchestration framework to address these challenges, thereby optimizing energy consumption, computational cost, network congestion, and service latency in dynamic IoT-MEC environments. The framework introduces task offloading and a dynamic resource orchestration strategy, where task offloading to the MEC server ensures an efficient distribution of computation workloads. The dynamic resource orchestration process, Service Function Chaining (SFC) for Virtual Network Functions (VNFs) placement, and routing path determination optimize service execution across the network. To achieve adaptive and intelligent decision-making, the proposed approach leverages Deep Reinforcement Learning (DRL) to dynamically allocate resources and offload task execution, thereby improving overall system efficiency and addressing the optimal policy in edge computing. Deep Q-network (DQN), which is leveraged to learn an optimal network resource adjustment policy and task offloading, ensures flexible adaptation in SFC deployment evaluations. The simulation result demonstrates that the DRL-based scheme significantly outperforms the reference scheme in terms of cumulative reward, reduced service latency, lowered energy consumption, and improved delivery and throughput.

## 1. Introduction

### 1.1. Background and Motivation

The Internet of Things (IoT) has been widely used for digitalization to expose the potential of utilizing network sensing and data gathering from citizen environments [1,2]. IoT device communication with several interface technologies (i.e., Wi-Fi, Bluetooth, ZigBee, LoRaWAN, NFC, and 6LoWPAN) through physical modulation [3,4,5] is used to access and communicate with network servers via fronthaul physical networks for diverse application scenarios. Thus, the increasing amount of data generated by IoT sensor networks leads to inefficient computation and resource utilization, resulting in the inability to conserve energy and reduce processing time on devices. IoT continues to generate tasks that require computing, with varying types of processing time, computing resources, and battery life. However, IoT generates massive traffic requests that often exacerbate bottlenecks and irregular fluctuations due to mobility and spatiotemporal variations in service usage patterns [6,7].

On the other hand, multi-access edge computing (MEC) can provide a solution for managing and orchestrating the resource utilization of computation from IoT sensing [8,9]. MEC has the potential to facilitate resource-intensive computation, which involves chaining virtualization resource capabilities and aims to reduce latency in communication protocols and network aspects [10,11,12]. However, MEC has the responsibility of addressing computational needs, leading to challenges in balancing execution delay and energy consumption. Additionally, the integration of MEC with software-defined networks/network function virtualization (SDN/NFV) controllers enhances the agility needed to reduce computation time and manage resource complexity, steering traffic patterns from ingress to egress. Service function chaining (SFC) provides instances of sequential virtual network functions (VNFs) to host virtual machines (VMs), allowing the order of VNFs to be propagated in the specified order for the required application. Consequently, MEC-enabled SDN/NFV offers lower latency and increased computational resources to manage the high demands of IoT services. Furthermore, the time needed to process resource allocation and placement for heterogeneous service functions poses a significant challenge for MEC, making real-time determination and adjustments essential for effective and efficient state transition decisions within this complex system.

**Figure 1:**
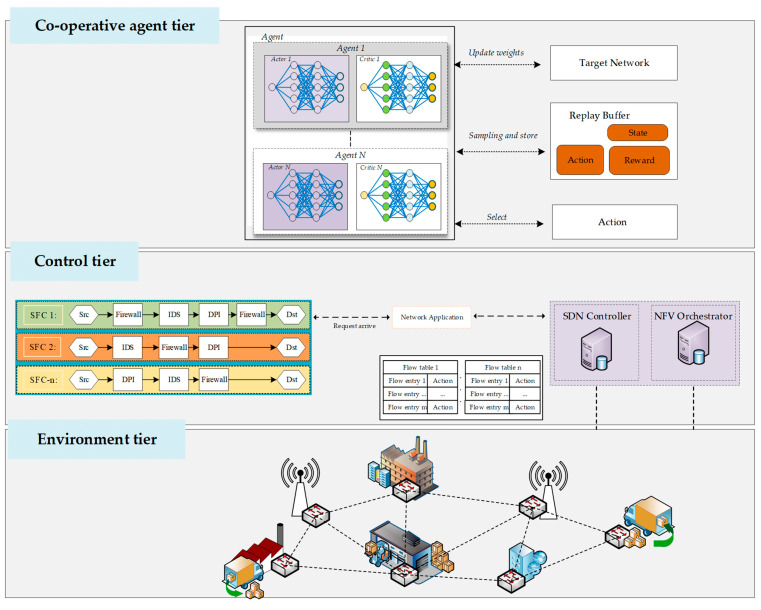
Multi-agent enabled in NFV for policy charging on resource utilization.

To address these limitations, Deep Reinforcement Learning (DRL) has gained attention as a powerful agent for making intelligent, adaptive decisions in highly dynamic networking [13,14]. By considering various system interactions, DRL-driven approaches can continuously learn from the network environment and SFC placements [15,16,17,18,19]. As shown in Figure 1, leveraging the DRL-integrated MEC framework can intelligently allocate VNFs to ensure SFCs are deployed to maximize resource efficiency, minimize latency, and maintain high reliability, even amid unpredictable traffic fluctuations as the workload changes.

Furthermore, the timely and efficient advancement of MEC and SFC technologies remains challenging due to the optimal placement and resource allocation of VNFs in IoT-driven network environments. On the other hand, they have delegated the computing capacities for real-time orchestration and resource management needed to instantiate VNFs to meet the workload. Moreover, the trade-offs between critical performance metrics, such as latency, energy efficiency, cost, and resource utilization, further complicate the issue. Achieving an optimal balance between these factors is difficult due to the limited resources available at edge nodes and the unpredictable demand from IoT devices. In traditional approaches, SFC deployment often relies on predetermined rules or heuristic methods that cannot adjust efficiently in real-time, leading to inefficient resource usage and poor service quality. As the scale of IoT networks continues to grow and the demands of applications become more complex, there is a clear need for intelligent, adaptive strategies that can dynamically optimize SFC placement, resource allocation, and workload distribution across the edge network. Moreover, DRL is used to determine the policy for charging SFC deployment in the MEC server, enabling adaptive allocation of optimal decisions for instance resources in fluctuating IoT networks.

### 1.2. Motivation and Contributions

This paper addresses these challenges by proposing a DRL-driven framework for intelligent and efficient SFC deployment in MEC environments, capable of learning optimal placement policies that adapt to fluctuating workloads and network conditions. The following recapitalization is the main contribution of this paper:

- We formulated the VNF placement and resource allocation problem as a multi-objective optimization task, where conflicting performance goals are considered to minimize latency, conserve energy, and optimize resource utilization.
- By intelligently distributing workloads across geographically distributed edge nodes based on real-time system states, the model ensures balanced resource utilization and reduces service degradation in high-density IoT deployments. Our scheme can proactively reallocate resources to avoid and minimize overload risks, thereby improving the long-term sustainability of edge nodes.
- We design a joint optimization algorithm for task offloading and resource allocation based on the Deep Q-Network, with the optimization objectives of assisting the NFVO in instantiating resources according to the workload requirements of IoT devices.
- The reward provides comprehensive network performance. Our proposed scheme leverages DRL frameworks and the network environment to highlight significant existing solutions across various aspects, including energy, latency, packet delivery ratio, packet drop ratio, and throughput.

### 1.3. Paper Organization

The remaining section of our study is organized as follows: Section 2 provides a literature review of existing work. Section 3 discusses network modeling using DRL to optimize the VNF resource allocation and placement. Section 4 outlines the performance metrics and compares the proposed methods with reference methods, concluding with a discussion in Section 5.

## 2. Related Work

In response to the current trend of utilizing IoT devices efficiently, many researchers have focused on resource and energy consumption to handle resource-intensive computing workloads. However, many studies investigating computing in the IoT network are still challenging due to the vast amount of big data and latency required to orchestrate resources and achieve real-time efficiency.

Many research scholars aim to optimize resource allocation and placement on MEC to enhance resource utilization concerning communication and computation-intensive resources [20,21,22]. Moreover, reducing latency for delay-sensitive tasks on MEC servers poses a significant challenge in this effort. Traditionally, resource allocation techniques rely on heuristic or optimization-based approaches, which struggle to adapt to the real-time and unpredictable nature of IoT traffic patterns. For example, centralized methods such as Mixed Integer Linear Programming (MILP) and convex optimization have been employed to optimize latency and bandwidth utilization [23,24]. In their work, Ref. [25] presented efficient task offloading and profit maximization in MEC-enabled 5G Internet of Vehicles (IoV). This study proposed a Lyapunov-Based Profit Maximization (LBPM) algorithm to optimize the time-averaged profit of MEC providers while ensuring timely and effective task execution from vehicles.

Additionally, the previous study on resource management in VNF placement still faces the problem of duplicates in the form of chaining and overwhelming resource utilization. For instance, refs. [26,27,28] study VNF placement and traffic routing simultaneously, dividing the methods into two phases: first, VNF placement, and then establishing the executing links. To achieve optimal resource utilization, ref. [27] consider the tradeoff between resource consumption and links, providing a two-phase VNF placement methodology that uses constrained depth-first search algorithms (CDFSAs) and path-based greedy algorithms to assign VNFs with minimum resource consumption [28]. The authors proposed two methods, hybrid SFC and a heuristic algorithm, to solve the dynamic SFC embedding for chaining VNF nodes. However, their utilization of the shortest path or greedy algorithms could not achieve network load balancing.

In recent years, some researchers have started applying machine learning to tackle various optimization problems [29,30,31], with a focus on resource allocation, resource placement, scheduling, traffic routing, resource offloading, and SFC orchestration. For instance, ref. [32] proposed PPO-ERA to provide a real-time, adaptive, and dynamic strategy for VNFs, addressing task delays and resource utilization. Meanwhile, SFC deployment over MEC servers shares idle computing resources that utilize the same SFC. In [33], VNF cooperative scheduling with priority-weighted delay is examined. Their work incorporated DRL with a target Q-network to enhance solutions for the optimal problem related to VNF scheduling, while supporting multidimensional resources in edge nodes. In [34], a multi-objective SFC mapping technique based on DQN is proposed to achieve delay and load balancing targets. In their work, Ref. [35] leverages the DQN and MQDR-based method for the dynamic deployment of customized SFCs, including dynamic adjustments of SFCRs. Furthermore, in [36], the VNF formulation problem is addressed using integer nonlinear programming to minimize bandwidth costs and ensure service execution for end-to-end delays.

Through their strategies and significant efforts, they enhance resource efficiency and effective resource utilization. However, computation and resource allocation to idle VNFs present challenges and drawbacks, particularly in terms of reallocating resources and placement. This can lead to overwhelming utilization in real time within the same SFC.

To clarify the significance of our research, we present the utilization of VNF resource placement and allocation approaches compared to the previous studies mentioned above. We develop a novel dynamic resource allocation and placement based on diverse SFR in NFVO. VNFs are monitored by NFVI to control resource adjustments that respond to real-time changes in VNFs within SFCs, coordinating with DQN variance methods. Furthermore, we consider the priority of SFC for managing resources based on workload in computing tasks.

## 3. Model and Problem Formulation

This system model section discusses our system simulation and represents the complete VNF and SFC related to MEC servers and the IoT sensing network. Regarding the resource allocation issue in the VNF of the SFC, the optimization goal of this paper is to address resource placement and facilitate cost-effective application deployments by considering the services required for managing end devices.

### 3.1. Network Model

In the network section, we examined the allocation and placement of NFV resource utilization to align with the computation and communication demands of MEC workloads. In this paper, we illustrate the physical infrastructure resource instantiation involved in creating several Network Functions Requests (NFRs) to support multiple Service Functions (SFs) in terms of requirements and monitoring resource workload. IoT contributes to resource-intensive network data traffic, which escalates resource-heavy computation and complex workloads in edge networks, as depicted in Table 1.

In the system model, the undirected graph is leveraged to represent network function resources, where G is a set of MEC servers and V is the subset of MEC nodes that connect to the link denoted as E. The task process can be defined in the primary phase as follows:

- Offloading task from IoT to MEC server for computing task: In each *timeslot-t*, IoT devices offload task−j to MEC−m. Equation (1) indicates the communication model that is associated between end-devices and the MEC-server for data rate, denoted as ${ED}_{n-m}^{t}$ for the state of allocated bandwidth ${bw}_{n\to m}^{t}$, channel gain $g_{n\to m}^{t}$, transmission power $p_{n}^{t}$, and noise ∀ℵ.

(1) $${ED}_{n-m}^{t}={bw}_{n\to m}^{t}\log_{2}(1+\frac{p_{n}^{t}g_{n\to m}^{t}}{\forall ℵ})$$

In this scenario, we assume that the IoT fully offloads the task−j to MEC −m for computation in every timeslot−t, and all the MEC servers are supposedly equipped in every single device.

### 3.2. SFC Requests

The set IoT-R is denoted by F. The j-th  IoT denotes IoT-$R_{j}$, responding to a 5-tuple ($S_{j},D_{j},\;P_{j},R_{bw,j,},R_{delay,j},R_{cpu,j}^{f}$). In this tuple, $S_{j}$ and $D_{j}\;$ represent the source IoT device and destination MEC server, respectively. The set $P_{j}$ denotes the sequence of VNF requirements by IoT-$R_{j}$. $R_{bw,j,}\;\mathrm{and}\;R_{delay,j}$ denote the bandwidth consumption required by IoT-$R_{j}$ and maximum tolerated E2E delay of IoT-$R_{j}$, respectively.

#### 3.2.1. Resource Constraints

We ensure sufficient MEC servers’ resource to host VNFs and bandwidth for handling all the IoT-$R_{j}$:

(2) $$\sum_{i\in J}\sum_{f\in F_{j}}R_{cpu,j}^{f}\cdot x_{j,f}^{m}\leq C_{re,m}^{t}\;\;\;\forall m\in G,\forall t\in T$$

where $R_{cpu,j}^{f}$ indicates CPU demand of VNF-f in request-j, and $C_{re,m}^{t}$ demonstrates the remaining resource capacity of MEC-m at time-t.

(3) $$\sum_{i\in J}y_{j}^{e}(t)\cdot R_{bw,j,}\leq C_{re,m}^{t}\;\;\;$$

where $y_{j}^{e}\left(t\right)\;$ indicates whether link e is traversed by IoT-R in request-j∈R and equals 1 if the traffic of IoT-R travers physical link; 0 otherwise.

#### 3.2.2. Delay Constraint

We use ${PD}_{j}$ to indicate the total propagation delay of IoT-R not exceeding the maximum total of service requests:

(4) $${PD}_{j}=\sum_{e\in E}y_{j}^{e}(t)\cdot d_{e}\leq R_{delay,j}\;\;\;\forall j\in J,\forall t\in T$$

### 3.3. Optimization Modelling Designs

Improve resource allocation and placement of VNF over the MEC server. This paper aims to improve the system to maximize successful placement resources in service requests while maintaining effectiveness by minimizing energy consumption and delay for each application under computational and networking resources constraints.

(5) $$Max\sum_{t=1}^{T}\sum_{j=J}\left(\sum_{f\in F_{j}}\sum_{v\in V}x_{j,f}^{v}(t)-\lambda_{1}.E_{j}-\lambda_{2}.{PD}_{j}\right)$$

s.t. (2)–(4)

#### 3.3.1. State Space

**Figure 2:**
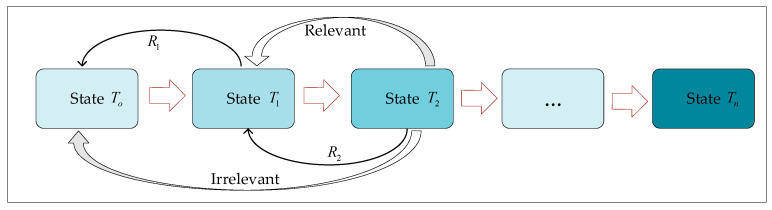
State space flow execution on an interval.

To address the network formulation for resource utilization in orchestration and management, the state is gathered from network components with critical states, as shown in Figure 2. The process proceeds by computing resource allocation, gathering resources from the network, and orchestrating for SFR. The Markov decision process is utilized to interact within the NFVO environment.

- $R_{m}$ represents the coordination of resources in the MEC server to preserve the ability to handle the computation tasks during IoT and offload the tasks to MEC-m.
- $C_{up,m}$ represents the upper-bound resource utilization in MEC-m at timeslot-t.
- BW is communication from the local device to MEC−m, state of total bandwidth, observed from a total bandwidth allocation and channel gain between environments.
- ${BW}_{up,m}^{t}$ represents how much bandwidth is allocated between nodes to MEC−m during the paging tasks at timeslot−t.
- A subset of a tuple $j_{j-m}^{comp}(t)$, which consists of processing tasks, including the consumed resources/energy, and time spent from experience.

(6) $$S_{t}=\left\{R_{m},C_{up,m},BW,{BW}_{up,m}^{t},j_{i-m}^{comp}(t)\;\right\}$$

#### 3.3.2. Action Space

The offloading task from IoT devices to the MEC server, whose backbone SDN controller has a global view of the flow entity, consists of the routing table synchronized as an action from the agent space. On the other hand, the mechanism of SDN-MEC enables DRL to empathize with the experienced batch of resource allocation and resource placement for performance patterns in each observation state iteration. $a_{j}^{off}\left(t\right)$ is set to determine the connection between IoT devices and MEC servers for offloading tasks. Additionally, $a_{mec|F}^{Plac}\left(t\right)$ is the optimal MEC selection for VNF placement to align the ordering of SFC forms in computing the task based on SFR. The corporation proposed by the agent and SDN controller is to optimize the flow rule of routing execution in installation and alleviation for the effective execution of tasks in terms of completion time within maximum tolerance delays.

(7) $$a_{t}=\{a_{j}^{off}\left(t\right),\;a_{mec|F}^{Plac}\left(t\right)\}$$

#### 3.3.3. Reward Based on Policy Charging Selection

In this proposed SDN-NFV, DRL aims to deal with the efficiency of applying the action $a_{t}$ into the MEC-IoT environment state $s_{t}$ by gaining the reward $r_{t}\;$ to show the impact of the action that transitions the following states: $s_{t+1}$. In this study, the primary reward by dented $R_{env}^{t}$ is to sum the total of two sub-rewards, reduced service latency and lowered energy consumption, denoted as $r_{Lat}^{t}\;and\;r_{Ene}^{t}$, respectively.

(8) $$R_{env}^{t}={⋋}_{lat}(t)\cdot r_{Lat}^{t}+{\;⋋}_{Ene}(t)\cdot r_{Ene}^{t}$$

where ${⋋}_{lat}(t)\;$ and ${⋋}_{Ene}(t)$ are time-varying weights (${⋋}_{lat}\left(t\right)+{⋋}_{lat}\left(t\right)=1)$ that adapt based on current conditions. When the network load is high or delay-sensitive service requests are prioritized, ${⋋}_{lat}(t)\;$ is increased to focus on reducing latency. Conversely, under lower loads or when energy conservation is critical,$\;{⋋}_{Ene}(t)$ is emphasized to minimize energy consumption.

The optimal policy is set to NFVO to orchestrate the resources from the overall batch that maximizes the long-term reward expectation by following Equation (9). On the other hand, Equation (10) presents an optimal policy selection for the state by following the Bellman equation.

(9) $${\pi \left(t\right)}^{*}=\underset{\pi }{\mathrm{argmax}}E_{\pi }\left[\sum_{t\in T}\gamma^{t}R_{env}^{t}\right]$$

(10) $$Q^{*}(s,a)=E_{\sim s^{\prime }}\left[R_{env}^{t}+\gamma \;\underset{a^{\prime }}{\mathrm{argmax}}Q^{*}(s^{\prime },a^{\prime })\right]$$

### 3.4. Pseudo DQN Algorithm Designs

To effectively address the dynamic and resource-constrained nature of IoT-MEC, we leverage DQN-based algorithms to ensure proper VNF placement and resource allocation utilization. In this section, Algorithm 1 presents the DQN algorithms with embedding of VNF resource placements. The DQN-based VNF placement algorithm operates in two phases: the learning phase and the execution phase. From the learning phase, input the state from the IoT-MEC environment, such as $\left(V,\;E,R_{m},\;C_{u,m},BW,{BW}_{up,m}^{t},C_{re,m}^{t}\;\left(t\right)\right)$. The algorithm begins by initializing the experience replay buffer and the online and target Q-network parameters. It then initializes the system state based on incoming SFRs, which include the resource requirements and the structure of the VNFFG. A batch of candidate MEC servers is sampled without repetition for potential VNF placement. At each timestep−t, a random value is generated to determine the action-selection strategy. If the value is greater than a threshold ϵ-epsilon, the algorithm selects an action randomly to encourage exploration. Otherwise, it chooses the action with the maximum Q-value, computed by the online network, to exploit the learned policy. The selected action involves deploying a VNF on a chosen MEC server and computing the shortest communication path within the VNFFG. The algorithm then observes the resulting reward and transitions to the next state, storing the experience tuple as a result $\left(s_{t},a_{t},r_{t},s_{t+1}\right)$ in memory from which mini-batches are randomly sampled during training. For each sample, the algorithm computes a target Q-value using the Bellman equation and updates the network parameters by minimizing the mean squared error between predicted and target Q-values. The network parameter update process includes both the online Q-network update via gradient descent and the periodic synchronization of the target network to ensure stable learning. Furthermore, time complexity, training involves the sampling of a batch of size N, with each forward and backward pass costing Ψ,  yielding O(N·Ψ) per training step; the inference, which only required going forward through the network, has a complexity O(Ψ) per decision step. The training is looped continuously until VNFs in SFR are successfully placed in order of requirement. In the execution phase, the trained Q-network is used to make placement decisions. The action with the maximum Q-value is selected and executed at each time step, updating the placement strategy and transitioning the environment state accordingly. This process repeats until the complete VNF chain is placed, at which point the algorithm outputs the final placement strategy $P_{MEC}^{j}$. This approach enables the algorithm to make efficient and scalable placement decisions in a resource-constrained MEC-NFV/SDN, thereby optimizing system-level performance metrics such as delay, energy consumption, and load balancing.

**Algorithm 1**: Pseudo-code of DQN-based VNF placement algorithms
**Input**


$\mathrm{State}=(V,\;E,R_{m},\;C_{u,m},BW,{BW}_{up,m}^{t},C_{re,m}^{t}\;\left(t\right))\;$


**Output**


$\mathrm{VNF}\;\mathrm{placement}\;\mathrm{and}\;\mathrm{allocation}\;\mathrm{in}\;\mathrm{MEC}\;\mathrm{servers}\;P_{MEC}^{j}$


**Learning process:**
1Initialize the experience reply.2
**For each**
3

Initialize the state s of VNF from SFR Sample a batch of MEC Server−m without repetition4

For each time step−t do5

Generate randomly value6
If random value, ≥ϵ **then**7



Randomly select an action a(t)

8

**Else**
9



Select of actions with the MAX Q−value

10

$a_{t}=a{rgmax}_{a_{t}}Q(s_{t},a_{t},\theta )$ based on online_net11

**End if**
12

Enforce the action that deploys VNF and calculates the shortest path of VNFFG13



$\mathrm{Calculate}\;\mathrm{the}\;\mathrm{reward}\;r_{t}$


$\;\mathrm{and}\;\mathrm{new}\;\mathrm{state}\;s_{t+1}$

14



$\mathrm{Store}\;\mathrm{the}\;\mathrm{state}\;\mathrm{transition}\;\mathrm{sample}\;(s_{t},a_{t},r_{t},s_{t+1})$

15



$r\left(s_{t},a_{t},s_{t+1}\right)+\gamma \;\underset{a_{t+1}}{\max}Q\left(s_{t},a_{t},\theta^{-}\right)$

16

$\mathrm{Get}\;\mathrm{updating}\;\mathrm{the}\;\mathrm{value}\;\mathrm{of}\;\theta^{-}=\theta \;$for every C steps17
**If** the VNF placement and deployment has yet completed;18

**Then**
20

**End if**
21
**End for**

**Execution process:**
22Read the online_net and target_net23

For each step−t

24

**Do**
25
Select the action with Max Q_value;26
Execute the action and update the placement scheme:27


$P_{MEC}^{j}=s_{t}$

28


$\mathrm{Update}\;\mathrm{the}\;\mathrm{state}:\;s_{t}=s_{t+1}$

29
**If** the VNF placement is completed, then.31

**End if**
32
**End for**
33
**Return**


## 4. Performance Evaluation

To conduct the network simulation [11,21], the network environment utilizes the NetworkX library to create network topologies for all network components. As depicted in Table 2, all simulations are executed on a computer with an AMD Ryzen (R) 7 5700X CPU, 3.0 GHz, 32 GB (Advanced Micro Devices, Inc., Santa Clara, CA, USA), NVIDIA RTX 4080 GPU (NVIDIA, Santa Clara, CA, USA), and Python programming. A network topology is instantiated to reveal the network for setting the network topology. Once the network-tolerant delay is set, the number of IoT devices is set to 100 to be attributed across five MEC servers. We utilize the DRL agent function with the OpenAI library within the DRL framework, initialized with PyTorch.

### 4.1. Comparison of Proposed and Reference Schemes

Our study conducts network evaluation and sets up three comparison schemes to illustrate how the proposed approach differs from the baseline approach. This comparison illustrates the performance differences in terms of various computation resource workloads for IoT-MEC task offloading, leading to enhanced energy efficiency and reduced overhead latency.

- DQN-MIoT is our proposed method, which utilizes DRL algorithms combined with NFVO in MEC to allocate resources for VNF placement. Our proposed DRL-based NFV control leverages a DQN architecture to approximate the Q-value function, enabling collaborative configuration of SDN/NFV flow rules for optimized placement and resource allocation. This method effectively trains the function approximator to manage high-dimensional state observations and representations. As detailed in Section 3, the approach incorporates experience replay, neural network training, and synchronization with the SDN/NFV controller to learn sophisticated policies.
- DQL-MIoT is set to leverage the Deep Q-learning approach, which enables learning from the network environment and applying action to control policy; however, DL achieves low performance in the complex network topology and can only handle lightweight network topology cases.
- Greedy-MIoT represents the traditional SDN/NFV for IoT-MEC servers in computing task management. This baseline approach addresses a centralized MANO that manages resources and controls the characteristics of IoT, adhering to the definition of standard NFV rules. The resource management controller is based on topology, traffic conditions, service requirements, and offloading policies. Greedy-MIoT relies on network-level optimization, efficient resource management, and resource allocation for non-complex applications.
- Random-MIoT indicates that the algorithm stochastically chooses an MEC service to an ingress source from traversing a VNF resource instance and a routing path to commonly chaining two adjacent VNF instances for every incoming IoT-R.

### 4.2. Results and Discussion

**Figure 3:**
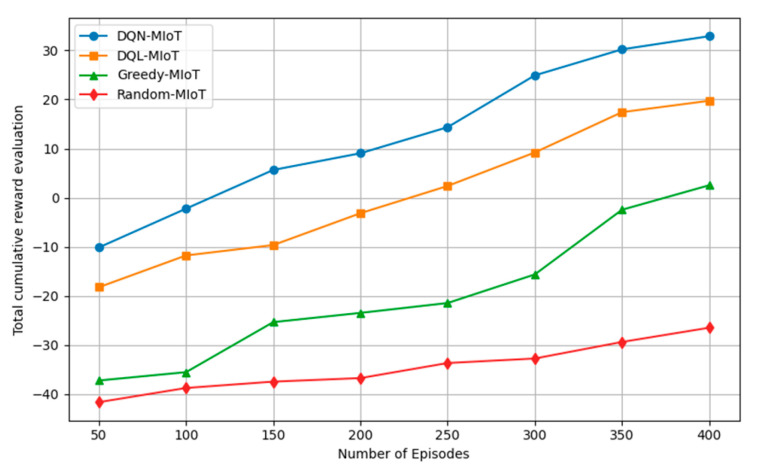
Result of cumulative reward evaluation.

In this section, we present the results for the proposed DQN-MIoT and reference schemes, namely DQL-MIoT, Greedy-MIoT, and Random-MIoT, in terms of cumulative reward, sub-rewards for latency and energy, packet drop ratio, packet delivery ratio, throughput, and delay in services. The measurement of this performance metric significantly showcases how our system setting integrates with the DQN framework, ensuring the reliability of chaining VNFs and adjusting for resource differences under diverse applications and workloads. The same topology and task size are used in this simulation, but the controller and agent differ in terms of performance metrics. The result demonstrates the total reward throughout each significant scheme’s DQN phase for exploration and exploitation within 400 episodes. Figure 3 illustrates the results of the proposed method, showing the total reward over 400 episodes, as defined in Equation (8). During the early learning phase (episodes 0–100), all approaches begin with negative cumulative rewards due to random policy exploration and ineffective action selection. As training progresses, DQN-MIoT shows a rapid improvement, achieving a positive cumulative reward after approximately 150 episodes, steadily increasing to 32.89 at episode 400. Meanwhile, DQL-MIoT gradually improves and achieves a final cumulative reward of 19.74, which is approximately 40% lower than DQN-MIoT. The Greedy-MIoT method shows minimal improvement, ending with a cumulative reward of only 2.53, which is approximately 92% lower than that of DQN-MIoT. The Random-MIoT baseline performs the worst, consistently maintaining negative rewards and ending at −26.48, indicating no effective learning or adaptation.

**Figure 4:**
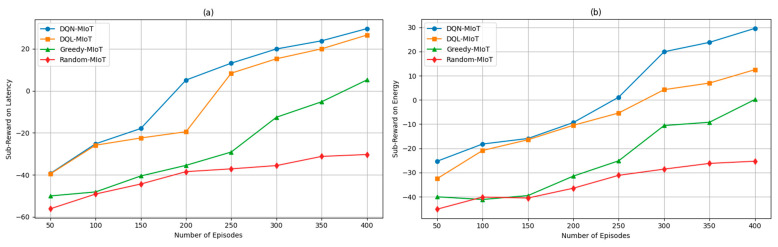
Performance metrics on sub-rewards of (**a**) latency and (**b**) energy.

In Figure 4a, initially, all methods exhibit negative sub-rewards, indicating poor latency performance due to random or sub-optimal policy actions during early learning phases. However, by episode 200, the DQN-MIoT approach begins to yield positive sub-reward values, signaling a successful adaptation to the MEC workload dynamics in IoT scenarios. As learning continues, DQN-MIoT and DQL-MIoT demonstrate robust convergence behavior, with DQN-MIoT consistently achieving higher sub-reward values than DQL-MIoT. By episode 400, DQN-MIoT achieves a latency sub-reward of 29.57, compared to 26.51 for DQL-MIoT, only 5.17 for Greedy-MIoT, and −30.31 for random-MIoT. This substantial gap underscores the benefits of employing a value-based Q-value with optimized exploration strategies for latency-sensitive SFC placement. Figure 4b illustrates the efficiency of energy score points as DQN-MIoT’s training process transitions the agent into a more stable policy regime, resulting in a higher immediate reward score. DQN-MIoT demonstrated a steady improvement, eventually peaking as the training progressed due to its adaptive learning strategy, which optimized energy consumption under network constraints. In contrast, DQL-MIoT showed more gradual progress, reflecting its slower convergence in handling energy efficiency within the MEC-enabled IoT environment. The Greedy-MIoT and Random-MIoT consistently underperformed with negative energy sub-rewards throughout most episodes, as they lacked adaptive mechanisms to manage resource allocation effectively. From the reward efficiency perspective, the proposed DQN-MIoT scheme dynamically adjusts resource allocation and offloading decisions, effectively balancing computational capacity and energy constraints. This adaptability enables improved handling of resource-constrained and computation-intensive tasks within the network, demonstrating strong suitability for MEC-enabled IoT scenarios where energy efficiency and task completion are crucial.

**Figure 5:**
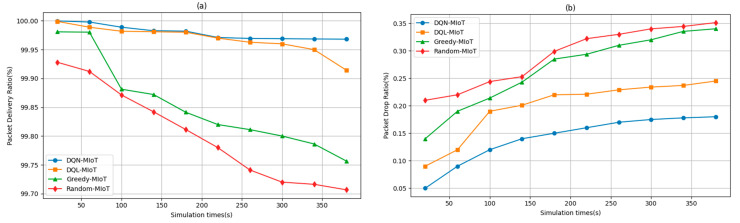
Performance metrics of (**a**) packet delivery and (**b**) packet drop ratio.

The results are demonstrated in Figure 5a,b for task delivery and drop ratios, where network states are configured consecutively by setting different network conditions, congestion levels, and application tasks every 400 simulation intervals. We evaluate the agent based on the fluctuation of investment results in the network parameter setting compared to the diversity of state observation. In high traffic fluctuations and computation-intensive environments, DQN-MIoT maintains its advantage with a PDR of 99.9681%, whereas DQL-MIoT drops to 99.9145%, and Greedy-MIoT declines further to 99.7568%. These results demonstrate the robustness and reliability of the DQN-MIoT framework in maintaining high data delivery rates even under increasing network loads. Meanwhile, at shorter simulation durations (20 s), DQN-MIoT achieves a notably low packet drop ratio of 0.05%, outperforming DQL-MIoT (0.09%), Greedy-MIoT (0.14%), and Random-MIoT (0.24%). As the simulation time increases to 380 s, DQN-MIoT maintains its performance advantage, recording a drop ratio of 0.18%, while DQL-MIoT, Greedy-MIoT, and random-MIoT reach 0.245%, 0.3401%, and 0.3513%, respectively. This consistent performance trend underscores the superior decision-making and adaptability of the DQN-MIoT framework in managing network traffic, confirming its suitability for real-time, data-sensitive applications.

**Figure 6:**
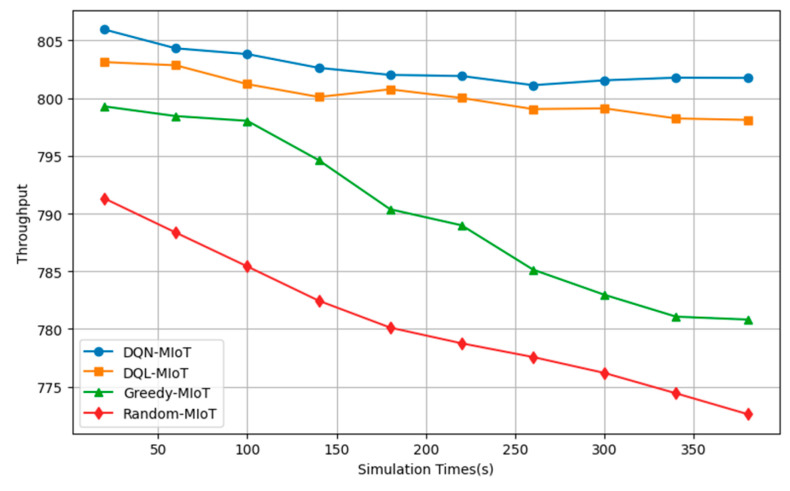
Result on throughput.

The throughput results presented in Figure 6, across different simulation times, further reinforce the superior performance of the DQN-MIoT approach compared to DQL-MIoT and Greedy-MIoT. At 20 s, DQN-MIoT achieves the highest throughput of 805.94 units, slightly surpassing DQL-MIoT (803.12) and notably outperforming Greedy-MIoT (799.28). DQN-MIoT maintains a stable throughput at 801.75, while DQL-MIoT, Greedy-MIoT, and random-MIoT drop to 798.11, 780.82, and 772.63, respectively. This steady trend indicates the DQN-MIoT model’s efficiency in handling data traffic under varying load conditions.

A thorough evaluation of performance metrics reveals that DQN-MIoT significantly outperforms reference schemes in terms of convergence speed, final reward value, and overall learning efficiency. Specifically, DQN-MIoT achieves a 66.7% higher final cumulative reward than DQL-MIoT, over 12 times higher than Greedy-MIoT, and vastly superior performance compared to Random-MIoT. This substantial improvement in DQN-MIoT’s strong ability to learn effective policies for balancing latency and energy trade-offs in dynamic MEC-enabled IoT environments leads to more intelligent and robust SFC deployment.

## 5. Conclusions

In this paper, we proposed the DQN-based NFV in IoT-MEC to handle resource computation for diverse services. We designed an intelligent task offloading and resource orchestration framework for dynamic IoT-MEC environments. The framework integrates SFC for VNF placement, dynamic route path selection, and workload distribution through MEC-based task offloading. By leveraging DRL, specifically a DQN-based Markov Decision Process, the system learns to make adaptive decisions for task offloading and resource allocation. Our simulation results indicate that DQN-MIoT outperforms baseline approaches in terms of sub-rewards on latency, energy consumption for MEC workloads within heavy fluctuating traffic changes, packet delivery ratio, packet drop ratio, and throughput in various network conditions and resource allocation values. In the future, we aim to incorporate memory-augmented and graph-enhanced learning models to overcome the limitations of LLMs in capturing long-term, multi-stage attack patterns. Moreover, future simulations will consider federated and edge-aware deployments to handle resource constraints in real-time environments. We will explore lightweight, explainable AI (XAI)-driven frameworks to improve interpretability and transparency in task orchestration.

## Figures and Tables

**Table 1:** Notation system model in the network model.

Notation	Description
G	Set of MEC servers
E	Set of links
V	Set of VNFs
J	A set of number tasks
N	The set of IoT devices
T	Number of timeslots
$R_{m}$	Resources of MEC servers
$P_{j}$	Set all the VNFs by *IoT-R_j_*
$C_{up,m}$	Upper-bound capacity of MEC servers on (CPU, RAM, Disk)
BW	Maximum of Bandwidths
$d_{e}$	Propagation delay between nodes on *link e*
F	Number of service requests
${BW}_{up,m}^{t}$	Upper-bound bandwidth of *MEC-m* at *timeslot-t*
$C_{re,m}^{t}$	Remaining resources of *MEC-m* at *timeslot-t*
	Decision variable
$x_{j,f}^{v}(t)$	Equals one if the VNF of the service request that is deployed at *node-v* at *timeslot-t,* otherwise
$y_{j}^{e}(t)$	Selecting the virtual link between *node-j* at *timeslot-t*, otherwise

**Table 2:** Parameter configuration in the network and DRL framework.

Parameters	Specifications
Hosting infrastructure	Ryzen(R) 7 5700x3d CPU @ 3.0 GHz, 32 GB, NVIDIA RTX 4080 GPU
Number of IoT	100
Number of MEC servers	5
Service request configuration	NF (types = 5)
	SFC type of length (2–5)
	Flow rate (64 Kbps–4 Mbps)
	Tolerable time (100–500)
Task complexity and sizes	Random set (Low, normal, high)—(256 Kbits, 512 Kbits, 1024 Kbits)
Learning rate	0.001
Discount factor	0.95
Batch size	Random set (32, 64, 128, 256)
Exploration	0.5
Number of episodes	400
Python platform	PyTorch

## Data Availability

Derived data supporting the findings of this study are available from the corresponding author on request.
